# Properties of Polyunsaturated Fatty Acids in Primary and Secondary Prevention of Cardiovascular Diseases in the View of Patients (Silesia, Poland)

**DOI:** 10.3390/nursrep12040094

**Published:** 2022-12-08

**Authors:** Karolina Krupa-Kotara, Mateusz Grajek, Agata Wypych-Ślusarska, Sandra Martynus-Depta, Klaudia Oleksiuk, Joanna Głogowska-Ligus, Elżbieta Szczepańska, Jerzy Słowiński

**Affiliations:** 1 Department of Epidemiology, Faculty of Health Sciences in Bytom, Medical University of Silesia in Katowice, 40-055 Katowice, Poland; 2 Department of Public Health, Faculty of Health Sciences in Bytom, Medical University of Silesia in Katowice, 40-055 Katowice, Poland; 3 Department of Human Nutrition, Faculty of Health Sciences in Bytom, Medical University of Silesia in Katowice, 40-055 Katowice, Poland

**Keywords:** cardiovascular disease, polyunsaturated fatty acids, eating habits

## Abstract

Background: Cardiovascular diseases are a major cause of morbidity and mortality in Europe. Lifestyle plays an important role in the primary and secondary prevention of cardiovascular diseases, apart from pharmacotherapy and diagnostics. Numerous studies confirm that the type and quality of fat consumed in the diet have a huge impact on the risk of cardiovascular diseases. Reducing the risk of cardiovascular disease can be helped by minimizing the proportion of saturated fatty acids in the diet and replacing them with polyunsaturated fatty acids. These acids and, above all, their long-chain forms have a positive effect on health. Aim: This study aims to assess the awareness of the properties of polyunsaturated fatty acids in the primary and secondary prevention of cardiovascular diseases in the opinions of patients of the Cardiology Department of the Racibórz Medical Center. Material and Methods: The analysis included 302 patients (113 women and 189 men) hospitalized in the Cardiology Department. The research method was the authors’ questionnaire consisting of the patients’ record and thirty closed questions. To answer the research questions posed and test the hypotheses, statistical analyses were carried out using the IBM SPSS Statistics version 25 package. Results: Among the respondents, the least frequently used healthy eating habit was the infrequent eating of fried foods. A total of 18.2% of respondents had such a habit. The most commonly used healthy eating habit was checking the fat content in products, which was performed by 67.2% of respondents. Among the respondents, 58.3% said that butter and margarine increase serum cholesterol. Conclusions: The analysis of the data shows that the place of residence, education, sex, and reason for hospitalization of the respondents did not affect the frequency of healthy eating habits. In addition, the subjects had a low amount of healthy eating habits.

## 1. Introduction

The main cause of increased morbidity and mortality in Europe is cardiovascular disease. For this reason and due to acute cardiovascular complications, about 45.8% of the country’s population dies annually in Poland [1,2,3]. In addition to pharmacotherapy and diagnostics, lifestyle is an important role in the primary and secondary prevention of cardiovascular diseases. The most important risk factor for the development of cardiovascular disease is inadequate diet. This is mainly related to the excessive consumption of fats and their inadequate composition. The diet of adults in Poland is based primarily on excessive consumption of highly processed products rich in saturated fatty acids. However, due to the functions performed by fats, they should not be completely excluded from the diet, but attention should be paid to the quantity and quality of fats consumed [4,5]. According to the Institute of Food and Nutrition (IŻŻ), the daily intake of fats should be 20–35% [6].

Numerous studies confirm that the type and quality of fat consumed in the diet have a huge impact on the risk of cardiovascular disease [1,2,3,4,5]. Consumption of saturated fatty acids can harm the lipid profile and increase the risk of developing cardiovascular disease. Even though they are the main source of energy in the body, excessive consumption of them raises the concentration of LDL cholesterol, increasing blood clotting, among other things. This, in turn, leads to the development of atherosclerosis and can cause ischemic heart disease. For this reason, their intake should be limited to 10% of daily energy requirements [6]. Reducing the risk of the onset and development of cardiovascular disease can be contributed to reducing the proportion of saturated fatty acids in the diet and replacing them with polyunsaturated fatty acids. These acids, and especially their long-chain forms, have been shown to have positive health effects. Consumption of fish and, therefore, omega-3 polyunsaturated fatty acids reduces the incidence of cardiovascular disease [7,8].

The ratio of fatty acids of the omega-3 to the omega-6 family is also extremely important; it is a determinant of the health-promoting properties of the diet. According to the recommendations for proper nutrition, it should be (4–5):1. An excessive supply of omega-6 fatty acids can lead to a reduction in the beneficial biological effects of omega-3 fatty acids. However, the current, common diet is characterized by an imbalance in the ratio of fatty acids consumed and excessive consumption of saturated fatty acids as well as too little intake of omega-3 fatty acids while consuming too much omega-6 fatty acids [9].

Prevention of cardiovascular disease is defined as activities aimed at both the general population, people with an increased risk of atherosclerosis and its complications, and those with symptoms of atherosclerosis. These activities are aimed at eliminating or minimizing the development of cardiovascular disease and the disability associated with it [10,11,12]. A properly balanced diet plays an overriding role in the prevention of atherosclerosis and its complications. Of greatest importance is replacing the energy provided by the consumption of saturated fatty acids with energy derived from unsaturated fatty acids and diversifying the daily diet with fish. The main goal of eating a properly fat-balanced diet is to lower serum LDL cholesterol levels and reduce the risk of cardiovascular incidents [12,13]. Secondary prevention applies to those with established coronary artery disease to reduce the risk of subsequent disease [14]. The mortality rate among post-MI patients is twice as high for about 10 years compared to the rest of the population. Having survived a myocardial infarction should mobilize the patient to make lifestyle changes, especially dietary changes. Studies of people after myocardial infarction have proven the benefits of polyunsaturated fatty acids [15]. A study showed that the use of supplementation with DHA and EPA acids reduced the overall mortality by 20% with cardiovascular-related mortality reduced by 30%, which is closely related to the antiarrhythmic properties of omega-3 fatty acids [16,17].

Lipids are the dominant factor in the development of atherosclerotic lesions. Omega-3 fatty acids, through their effect on lipid metabolism, are an important factor in the antiatherosclerotic effect. When used daily, doses of 2–5 g can reduce serum triglyceride concentrations by up to about 30%. Such effects are responsible for increased catabolism and reduced low-density lipoproteins (VLDL) production. Acids from this family minimally raise the concentration of LDL cholesterol and may also raise the concentration of the HDL fraction. Omega-3 PACs contribute to the reduction of postprandial lipemia by activating lipoprotein lipase, the enzyme responsible for the catabolism of very low-density lipoproteins (VLDL) and chylomicrons. The antiarrhythmic effect of omega-3 fatty acids has been widely reported. According to many studies, omega-3 fatty acids directly affect the electrophysiological processes of cardiomyocytes. The action on sodium channels shifts the state of dynamic equilibrium inactivation towards hyperpolarized potentials. The consequence of this action is that cardiomyocytes are less susceptible to excitation. In turn, the anticoagulant effect of omega-3 fatty acids is related to their antiplatelet properties. The mechanism involves inhibiting the formation of prothrombotic factors such as TXA2, interleukin 1 (IL-1), lipoprotein (a), and platelet-activating factor. Omega-3 fatty acid supplementation has been proven to increase the levels of prostacyclins that inhibit platelet aggregation [18,19,20,21].

Natural sources of EPA and DHA are algae, phytoplankton, fish fat, and also other marine organisms that feed on plankton and fish. The most important sources of omega-3 fatty acids are oily marine fish and seafood. Fish oils, compared to vegetable oils, are characterized by a lower content of omega-6 fatty acids, which is beneficial for maintaining a normal omega-6/omega-3 ratio [22]. An excess supply of omega-6 acids relative to omega-3 can reduce the beneficial effects of EPA and DHA [16]. The highest levels of omega-3 fatty acids distinguish mackerel fish (tuna, mackerel), herring fish (herring, sardines), salmon fish (trout, salmon), and anchovy fish (anchovies). The species of fish, physiological state, fishing period, and also nature of existence determine the content of omega-3 acids. In addition, rapeseed oil is a valuable source of acids from the omega-3 family [6]. Rich sources of a-linolenic acid (ALA) are vegetable oils (flaxseed, safflower), nuts (walnut, hazelnut, Brazilian), and pumpkin seeds. The main sources of linoleic acid (CLA), which belongs to the omega-6 family, are vegetable oils. The content in grape seed oil ranges from 58–78%, in soybean oil 48–59%, and in safflower oil 68–83% while in canola oil, it accounts for 15–30%. Egg yolk and also meat and meat products are a source of arachidonic acid (ARA). Another source of EPA and DHA supplements in the form of fish oil or capsules. Supplementation is aimed at people whose concentration of omega-3 fatty acids in the body is too low. Sources of omega-3 fatty acids for vegetarians and people allergic to fish protein can be algae, vegetable oils, or nuts [23,24,25,26,27].

Based on the standards of the Institute of Food and Nutrition, the adequate intake (AI) of linoleic acid (LA) is 4% of dietary energy; for a-linolenic acid (AL), the standard was set at 0.5%. The standards for LA and ALA were set based on the lowest average intake of these acids in various population groups of European countries for which no symptoms of deficiency of these acids were found [6]. Numerous studies have shown that regular consumption of fish or supplementation with omega-3 fatty acids contribute to a reduction in mortality from cardiovascular disease. Because of this, the standard AI (adequate intake) level for the intake of EPA and DHA acids in adults is 250 mg [6]. According to the American Heart Association (AHA), patients suffering from ischemic heart disease should consume about 1 g of EPA and DHA each day. As part of the prevention of cardiovascular disease, healthy people are advised to consume oily marine fish about twice a week and eat products that are sources of a-linolenic acid in the amount of 1.5–3 g. The best solution is to provide these acids in the form of fish. Supplementation of these acids in the form of capsules is also acceptable but under the constant supervision of a doctor. Supplementation of DHA and EPA acids in the form of capsules is necessary for patients who need to lower triglycerides [28]. According to the recommendations of the International Society for Study of Fatty Acids and Lipids, the energy from LA intake is 2% while ALA was 0.7%. According to the recommendations, the amount of eicosapentaenoic acid and docosahexaenoic acid in the diet should be 0.65 g and 0.5 g per day, respectively [6]. Current standards in Poland have set the daily energy requirements of omega-6 acids at 5–8%, and the level of omega-3 acids has been set at 1–2% [6].

An extremely important element in the primary and secondary prevention of cardiovascular disease is the use of a well-balanced diet. Numerous observational and experimental studies have shown that the type of fat provided by a diet plays a significant role in the prevention of cardiovascular disease. A diet high in saturated fatty acids significantly affects the lipid profile, thus contributing to the development of cardiovascular disease. For this reason, an appropriately balanced diet should be based on limiting the supply of saturated fatty acids while increasing the supply of acids from the omega-3 and omega-6 families. To achieve a cardioprotective effect, an appropriate quantitative composition and proper proportions of the fatty acids consumed are necessary [6,22,23,24,25,26,27,28]. Therefore, the main objective of this study is to evaluate the awareness of the properties of polyunsaturated fatty acids in the primary and secondary prevention of cardiovascular diseases of patients of the Cardiology Department of the Racibórz Medical Center.

Specific objectives of the study:

1. Evaluate the dietary habits of patients hospitalized in a cardiac unit.
2. Evaluate the relationship between place of residence, education, gender, diet and reason for hospitalization, and patients’ eating habits.

## 2. Materials and Methods

### 2.1. Study Design and Eligibility Criteria

During the study period from 25 November 2019 to 31 January 2020, 302 patients (including 113 females and 189 males) were hospitalized in the Cardiology Department of the Racibórz Medical Center. Written permission from the facility and authorization to process personal data were obtained. The mean age was 50 ± 11.3 (21–71) years. The respondents were informed about the purpose of the study, the use of the obtained results for scientific purposes only, and their anonymity. The characteristics of the study group are shown in Table 1.

The selection of the sample was purposive. The following inclusion criteria were considered: undergoing hospitalization in a cardiology department, age ≥ 18 years, health condition that allows you to take part in the study, and giving written consent to participate in the study.

Patients received the questionnaire in paper form upon admission to the ward, provided that their health condition allowed them to complete it, and they gave informed written consent to complete it, which resulted in such a distribution of age and other variables in the study population.

The survey was conducted using a direct survey method with confidentiality maintained. All data were coded with appropriate symbols to prevent patient identification by the law of 29 August 1997 on the Protection of Personal Data (Journal of Laws 1997 No. 133 item 883).

The study design, in light of the act of 5 December 1996 on the professions of physicians and dentists (Journal of Laws of 2011, No. 277. item 1634, as amended), is not a medical experiment and does not require evaluation by the Bioethics Committee of the Silesian Medical University in Katowice, as it is based on the patient’s own experience. In addition, the data collected was based on an anonymous questionnaire to which patients gave written, voluntary consent. The questionnaires were distributed directly to the patients, so the absence of a completed questionnaire also meant that the patients did not agree to participate in the study. The study was conducted according to the principles of clinical research based on the Declaration of Helsinki, as amended.

### 2.2. Study Procedure and Research Tool

A proprietary questionnaire was prepared to conduct the study (See in Supplementary Materials). The questionnaire consisted of metrics including questions about the year of birth, weight, height, gender, education, social status, and the reason for hospitalization in a cardiac unit. In addition, the questionnaire included thirty closed-ended questions on the properties of polyunsaturated fatty acids in the primary and secondary prevention of cardiovascular disease. Subjects were instructed in detail on how to complete the survey. The questionnaires were reviewed for correct completion and carefully analyzed. Based on the completed metric (weight and height), body mass index (BMI) was calculated. Interpretation of the results obtained was based on the WHO classification of BMI values.

The survey was conducted in three stages. The first stage was a pilot study, during which 20 respondents were asked to fill out a questionnaire to check for understanding of all questions. Most of the questions were found to be clear and understandable to the respondents while questions that were indicated by at least 2 respondents as incomprehensible or unclear were removed or corrected. Stage two was the validation of the questionnaires by distributing them twice to a group of 50 respondents. An interval of 2 weeks was maintained between colocations of the questionnaires. The consistency of answers to the same questions was checked. For the reproducibility of the results obtained by the questionnaire used, the value of the κ (kappa) parameter was calculated for each question in the questionnaire—for 73.8% of the questions, very good (κ ≥ 0.80) method consistency was obtained while for 26.2% of the questions, good (0.79 ≥ κ ≥ 0.60) method consistency was obtained. The final stage of the study was to conduct the actual survey.

### 2.3. Statistical Analyses

Statistical analyses were carried out using the IBM SPSS Statistics version 25 package, with which an analysis of basic descriptive statistics and a series of univariate analyses of variance were performed. The classical threshold of α = 0.05 was considered the level of significance; however, the results of probability test statistics at the level of 0.05 < α < 0.1 were interpreted as significant at the level of the statistical trend.

## 3. Results

In the first step, the healthy eating habits of the subjects were checked, assuming that more healthy habits would correlate with higher awareness. One point was awarded for each answer indicating healthy habits while zero points were awarded for the others. Thus, respondents could receive a minimum of zero points (no healthy eating habits at all) and a maximum of seven points (completely healthy eating habits). The most common score in the sample was two points, which indicates a very low amount of healthy eating habits. Next, it was checked in which questions the respondents most often showed healthy eating habits and in which the fewest responses indicated adequate habits. As can be seen in Table 2, the least frequent healthy habits included the question about the frequency of eating fried foods, where only 18.2% of respondents answered that they do not eat such dishes. Moreover, only 19.2% consumed fish twice a week, and 19.5% of respondents chose lean cottage cheese. On the other hand, the most common healthy habits included checking the fat content of products, which was done by as many as 67.2% of people, and recognizing that butter and margarine raise cholesterol, which was marked by 58.3% of people.

Based on BMI, 34.8% of patients were normal weight (BMI 18.5–24.9 kg/m^2^), 37.1% were overweight (BMI 25.0–29.9 kg/m^2^), and 28.1% were obese (BMI > 30.0 kg/m^2^). There were no underweight patients in the group. About 67% of the overweight and obese patients were male and 33% female (Table 3).

Whether the place of residence is related to healthy eating habits was tested. One-way analysis of variance with the calculation of the omega2 measure was used for calculations. Table 4 shows the results on the healthy eating scale by place of residence.

The results indicated that place of residence was not significant (*p* = 0.928) for the occurrence of healthy eating habits. Respondents obtained similar results regardless of their place of residence.

After verifying the association of healthy eating with education, a one-way analysis of variance was used to see if education correlates with the number of healthy eating habits. Table 5 shows the results of the calculations.

It turned out that the relationship between education and healthy eating was at the level of statistical trend and had weak strength. Post hoc tests comparing the number of healthy eating habits were then performed. However, the analyses showed no statistically significant differences between the pairs (*p* = 0.098).

A *t*-test for independent samples was used to test the gender difference in healthy eating. The strength of the difference was determined using Cohen’s d measure. The results are shown in Table 6.

The results indicated that there was no statistically significant difference in healthy eating between men and women (*p* = 0.323). Respondents, regardless of gender, had a similar number of healthy eating habits.

Finally, calculations were performed to determine whether the association between the reason for hospitalization and healthy eating habits was statistically significant. A one-way analysis of variance was again used to verify the association. Table 7 presents the results obtained.

Moreover, the reason for hospitalization was not statistically significant for the number of healthy habits (*p* = 0.810). Patients with different reasons for hospitalization scored similarly on the healthy eating scale.

## 4. Discussion

The beneficial effects of polyunsaturated fatty acids on the cardiovascular system have been confirmed by numerous observational and experimental studies [29]. The results of these studies confirm the protective properties of polyunsaturated fatty acids on the cardiovascular system. Dietary habits play a significant role in the primary and secondary prevention of cardiovascular diseases [30]. Therefore, it is important to pay attention to the quality and type of fats supplied with the diet. In both the prevention and treatment of cardiovascular disease, it is necessary to follow an appropriate diet. The diet used should contribute to normalizing body weight. An indispensable element in the prevention of cardiovascular disease is to maintain body weight with a BMI in the range of 20–25. In addition, recent studies have shown that following a diet as close as possible to the Mediterranean diet reduces the risk of a cardiovascular incident [31]. A study by Schroeder et al. found that following a Mediterranean diet or habits similar to the principles of the Mediterranean diet negatively correlates with the occurrence of obesity [32].

According to the Position Statement of the Polish Dietetic Association, a controlled fatty acid diet is recommended for primary and secondary prevention and treatment of cardiovascular disease. Dietary fat supply should be limited to below 30% of total energy. Saturated fatty acids should not exceed 7% of dietary energy while cholesterol supply should not exceed 300 mg/d. The recommended supply of EPA and DHA acids is 250–500 mg/d. The source of these acids in the diet should be regularly consumed fish. According to PTD recommendations for cardiovascular prevention, two servings of fish should be consumed per week; each serving should weigh 140 g. At least one of the servings should be of an oily marine fish species [33].

For people postmyocardial infarction, the National Institute for Health and Care Excellence (NICE) recommends a supply of at least 7 g of omega-3 fatty acids per week. The source of these acids should be fatty fish in the amount of two to four servings per week. For those who do not consume the recommended amounts of fish, a supply of 1 g of omega-3 fatty acids in supplement form is recommended [34]. According to the Food Standard Agency’s recommendations, the maximum consumption of oily fish per week is four servings (excluding pregnant and lactating women, for whom two servings are recommended). Other recommended sources of omega-3 fatty acids include plant-based products such as soybean oil, canola oil, flaxseed oil, and also walnuts [35].

Unambiguous research results confirm a negative correlation between nut consumption and the risk of cardiovascular disease. Nuts are a highly nutritious source of unsaturated fatty acids. The PREDIMED study showed that a Mediterranean diet enriched each day with a serving of 30 g of nuts plays a beneficial role in the prevention of cardiovascular disease [36].

This survey conducted with patients hospitalized in the Cardiology Department shows that the respondents were characterized by a low number of healthy eating habits. The analysis of the data shows that the most common bad eating habit among the respondents was the consumption of fried foods. This habit affects 81.8% of the respondents. The method of thermal processing of prepared foods is extremely important in the prevention of cardiovascular diseases. The process of frying contributes to the fat content of a given dish or product. The proper technique for preparing meals and dishes is boiling water and steaming or braising food without adding fat using Teflon pans. To ensure that the food being prepared does not absorb extra fat, it can also be prepared on ceramic cookware, combi-brewers, on a grill, and also using an oven sleeve or transparent foil. Another bad habit relates to the frequency of eating fish. Among the respondents, 80.8% admit that they eat fish much less frequently than recommended. A sizable percentage of respondents (80.5%) answered that they choose semiskim or fatty cottage cheese instead of lean cottage cheese, which does not follow the principles of proper nutrition. Choosing cottage cheese with a higher fat content makes it difficult to reduce the amount of fat in the dietary ratio. Among respondents, 70.5% admitted that they do not use the recommended canola oil for frying. Respondents were more likely to use margarine and butter. For short frying, oils high in monounsaturated fatty acids and low in polyunsaturated fatty acids are suitable. The recommended oil for frying is canola oil. In its composition, it contains 61% monounsaturated fatty acids while the content of polyunsaturated acids is 29% [37,38]. The diet of 65.9% of respondents was dominated by animal fats. Only 58.3% of the respondents thought that consuming butter and margarine in excessive amounts can cause elevated serum cholesterol levels. The remaining 41.7% of respondents took the opposite view. In the prevention of cardiovascular disease, products that are a source of saturated fatty acids, which contribute to an increase in the concentration of atherosclerotic cholesterol, should be limited. Such products include butter, margarine, cream, lard, fatty cheeses, and fatty meats. As many as 32.8% of respondents do not pay attention to the fat content of the product they choose. Because of the need to make changes in their current diet, it is necessary to check the fat content on product labels and carefully analyze them. A study by Wegrowski et al. [39] confirms that most patients with cardiovascular diseases are characterized by inappropriate eating habits.

In the study, the relationship between place of residence and eating habits was analyzed. The data showed that the place of residence did not affect the eating habits of the respondents. Analysis of the study conducted by Babiarczyk [40] also did not confirm a statistically significant relationship between place of residence and the eating habits of respondents. In contrast, a study by Bieniasz et al. [41] found statistically significant differences between eating habits and place of residence. Respondents living in cities were more likely to have normal eating habits.

The study analyzed the relationship between education and the eating habits of the respondents. None of the analyses conducted confirmed the relationship studied. A study by Wegrowski et al. [39] found that people with primary, vocational, secondary, and higher education have comparable eating habits. A study by Babiarczyk and Dudek [40] also failed to confirm the relationship between the subjects’ education and eating habits.

Another relationship analyzed in the self-reported study concerned gender and eating habits. The results of the analyses do not confirm a gender difference on the subject of eating habits. Respondents, regardless of gender, were characterized by similar eating habits. Different results were obtained in a study conducted by Babiarczyk and Dudek [40]. The results of that study confirmed the relationship between gender and the eating habits of the respondents. In addition, women showed significantly more correct eating habits than men [40]. Women were characterized by higher scores in the category of correct eating habits compared to men [40].

The last relationship analyzed was between the reason for hospitalization and the eating habits of the respondents. This relationship was also not confirmed. Patients hospitalized for different reasons were characterized by similar eating habits.

In conclusion, the survey shows that patient awareness of the properties of polyunsaturated fatty acids in the primary and secondary prevention of cardiovascular diseases is too low. Therefore, it is necessary to conduct health education so that patients acquire the right information and skills to make the right health decisions. This is related to the inseparable making of informed decisions that concern health based on scientifically proven knowledge [42]

Health education methods focus on the patient and his or her coping skills, understanding shared responsibility for health and the impact of health behaviors, and the need to modify them. Health education in which various methods and didactic forms are used contributes to improving the level of patients with cardiovascular diseases [42]. An important role in the formation of proper eating habits among patients with cardiovascular diseases is played by medical personnel. His role should include planning and implementing individual nutrition. A multidisciplinary team including a doctor, nurse, and dietician can develop diet and nutrition recommendations and then supervise the implementation of the nutrition plan. In addition, they shape correct eating behavior among patients by selecting appropriate products and foods and using appropriate thermal processing methods. Health promotion and health education carried out by medical personnel is the most effective way to increase patient knowledge and reduce mortality from cardiovascular diseases [43].

## 5. Strengths and Limitations

The results of the present study have important utilitarian significance. Pointing out the decidedly inappropriate dietary habits of the patients surveyed, they underscore the need for extensive programs and public campaigns that take dietoprophylaxis into account. The importance of balanced nutrition in diseases of civilization is an indisputable fact. In Poland, however, it seems that nutrition education programs need broader outreach, as they are directed primarily to children and adolescents, young people, and to a lesser extent to the elderly. It is also not out of the question that this group is less receptive to nutrition education due to their previous, often incorrect, dietary habits and patterns. Therefore, the results of the present study make us reflect on the condition, reach, and addressees of dietoprophylaxis.

This study focused on examining awareness of the properties of polyunsaturated fatty acids in the primary and secondary prevention of cardiovascular disease. Other dietary behaviors were not analyzed. On the one hand, this may seem to be a certain limitation of the study but only in appearance. This was a deliberate intention of the authors for reasons regarding the dietary traditions in Poland where, for a long time, mainly fats of animal origin (mainly lard, butter, cream) were consumed, and frying was the basic technique of processing products. Thus, the results of the present analysis identify an area that requires nutrition education or re-education.

Limiting the study to patients with a specific disease entity may not allow for a random sample, so it is difficult to get a representative group, which may lead to bias in the study in some cases, but the authors have made every effort to reduce the risk of error. In addition, the inclusion criteria used did not allow for the inclusion of all people in the study due to health status or age.

The subject under consideration is in line with the strategic and operational objective of the National Health Program for 2021–2025, which is an implementing act of the law of 11 September 2015 on public health, as a strategic response to the need to counteract negative epidemiological trends and the increasing burden of chronic noncommunicable diseases on the population, among which cardiovascular diseases are classified.

This study on the awareness of primary and secondary prevention of cardiovascular diseases in populations already burdened will allow a better definition of the health needs of patients and proper targeting of activities regarding prevention, jurisprudence, social support groups, and thus more effective assistance and reduction of social and economic costs of cardiovascular diseases.

The timing of the study is an undoubted advantage. It was a period before the announcement of the pandemic, so the influence of the SARS-CoV-2 virus on possible cardiovascular complications can be excluded, and thus these circumstances can be ruled out as a cause of hospitalization.

The limitations indicated, however, do not diminish the importance of this study. Indeed, it reveals a significant problem associated with poor eating habits and points to the need for in-depth and nationwide nutrition prevention programs.

## 6. Conclusions

Based on the study, it was concluded that the awareness of the properties of polyunsaturated fatty acids in the primary and secondary prevention of cardiovascular diseases among patients of the Cardiology Department of the Racibórz Medical Center was insufficient. In addition, the study concluded that:

1. Patients hospitalized in the cardiac unit were characterized by low levels of healthy eating habits.
2. There was no relationship between place of residence, education, gender, or reason for hospitalization and patients’ eating habits.

## Figures and Tables

**Table 1:** Characteristics of the study group (*n* = 302).

Variable	*n* (%)
**Sex**	
Female	113 (37.4)
Male	189 (62.6)
**Age**	
21–28	1 (0.3)
29–38	15 (4.9)
39–49	46 (15.3)
50–71	240 (79.5)
**BMI**	
Obesity	85 (28.1)
Overweight	112 (37.1)
Normal weight	105 (34.8)
Underweight	-
**Reason for hospitalization**	
Shortness of breath	48 (15.9)
Chest pain	39 (12.9)
Myocardial infarction	164 (54.3)
Heart failure	11 (3.6)
Heart rhythm disturbances	27 (8.9)
Uncontrolled hypertension	13 (4.4)
**Comorbidities**	
Type 2 diabetes	178 (58.9)
Hypertension	267 (88.4)
Atherosclerosis	223 (73.8)
Chronic kidney disease	103 (34.1)
Chronic obstructive pulmonary disease (COPD)	86 (28.8)
Heart failure	167 (55.3)
**Education**	
Primary	17 (5.6)
Vocational	176 (58.3)
Secondary	49 (16.2)
Higher	60 (19.9)
**Place of residence**	
Village or city of up to 20 thousand residents	128 (42.4)
City of 20 thousand–100 thousand residents	118 (39.1)
City of more than 100 thousand residents	56 (18.5)

**Table 2:** Share of responses indicating healthy eating habits and lack thereof.

Variable	Lack of Healthy Eating Habits		Healthy Eating Habit	
	*n*	%	*n*	%
Paying attention to the fat content of products	99	32.8	203	67.2
Recognition that butter and margarine raise cholesterol	126	41.7	176	58.3
Type of fat dominant in the diet	199	65.9	103	34.1
Reaching for lean cottage cheese	243	80.5	59	19.5
Used fat for frying food	213	70.5	89	29.5
Frequency of consumption of fried foods	247	81.8	55	18.2
Frequency of fish consumption	244	80.8	58	19.2

**Table 3:** WHO classification of patients’ BMI values.

BMI (kg/m^2^)	WHO Classification	BMI of Patients *n* (%)
<18.5	Underweight	-
18.5–24.9	Standard	105 (34.8)
25.0–29.9	Overweight	112 (37.1)
30.0–34.9	first-degree obesity	78 (25.8)
35.0–39.9	secondary obesity	4 (1.3)
≥40	tertiary obesity	3 (1.0)

**Table 4:** Relationship of healthy eating to a place of residence.

Variable: A Place of Residence	Healthy Eating		
	M	*SD*	*n*
Village	2.4	1.19	128
City of 50,000 to 100,000 inhabitants	2.44	1.2	118
A city with more than 100,000 inhabitants	2.46	1.09	56
	*F*	*p*	ω^2^
The relationship between healthy eating and where you live	0.07	0.928	0

**Table 5:** Relationship of healthy eating with education.

Variable: Education	Healthy Eating		
	M	*SD*	*n*
Primary	3	0.94	17
Professional	2.47	1.17	176
Medium	2.33	1.23	49
Higher	2.33	1.16	60
	*F*	*p*	ω^2^
The link between education and healthy eating	2.12	0.098	0.01

**Table 6:** Relationship of gender to healthy eating.

Variable: Gender	Women (*n* = 113)		Men (*n* = 189)				95% CI		*d* Cohen’s
	M	*SD*	M	*SD*	*t*	*p*	*LL*	*UL*	
Healthy eating	2.51	1.28	2.38	1.10	0.99	0.323	–0.14	0.41	0.12

**Table 7:** Association of healthy eating with the reason for hospitalization.

Variable: The Reason for Hospitalization	Healthy Eating		
	M	*SD*	*n*
Shortness of breath	2.54	1.17	48
Chest pains	2.23	1.04	39
Myocardial infarction	2.41	1.26	164
Heart failure	2.63	1.03	11
Heart rhythm disturbance	2.56	0.89	27
Uncontrolled hypertension	2.38	1.12	13
	*F*	*p*	ω^2^
Relationship between healthy eating and reason for hospitalization	0.46	0.810	0

## Data Availability

Not applicable.

## Supplementary Materials

The following supporting information can be downloaded at: https://www.mdpi.com/article/10.3390/nursrep12040094/s1, Questionnaire.
