# Supplementary material for: Properties of Polyunsaturated Fatty Acids in Primary and Secondary Prevention of Cardiovascular Diseases in the View of Patients (Silesia, Poland)
Source: Nurs Rep. 2022 Dec 8;12(4):980–92. doi: 10.3390/nursrep12040094 (PMC9786799; doi:10.3390/nursrep12040094)
Supplement: Supplementary file 1 [file nursrep-12-00094-s001.zip › nursrep-2023640-SI.pdf]

Appendix 2

The presented questionnaire was translated for illustrative purposes. The original version of the questionnaire for research purposes was used in Polish. The questionnaire is submitted as an appendix to an article reported in the journal Nursing Reports.

QUESTIONNAIRE

Dear Sirs and Madame,

I kindly request your permission to participate in this study. The purpose of this study is to assess *awareness of the properties of polyunsaturated fatty acids in the primary and secondary prevention of cardiovascular disease.*

The present research is conducted for the purpose of a thesis carried out at the Silesian Medical University in Katowice. The questionnaire is **anonymous** and the results will be used for scientific purposes only.

Please answer honestly and completely, as the value of the survey depends on it. Please mark with "×" the selected answer or provide information in the designated spaces and do not skip any questions.

The answers are **single-choice**, i.e., you choose only one answer option in each question unless the question content asks you to select more than one of the answers.

Statement

I give my informed consent to participate in the study by completing the questionnaire

YES ☐

NO ☐

I sincerely thank you for your time.

METRIC

Year of birth ....

Body weight [kg] ....

Height [cm] ....

Gender

a) woman
b) man

Education

a) basic
b) professional
c) average
d) higher

Place of residence

a) village
b) city of 50,000 to 100,000 inhabitants
c) city of more than 100 thousand inhabitants

Professional status

a) pupil/student
b) active person
c) non-worker
d) pensioner
e) pensioner(s)

Social status

a) miss/cohabitant
b) single
c) married
d) divorcee/divorcee
e) widower/widow

How do you assess your health condition?

a) very good
b) rather well
c) on average
d) rather bad

Reason for your hospitalization in the cardiology department

a) myocardial infarction
b) heart failure
c) uncontrolled hypertension
d) cardiac arrhythmias
e) dyspnea
f) chest pains

Are you accompanied by such diseases as:

a) diabetes

b) hypertension
c) atherosclerosis
d) chronic kidney disease
e) chronic obstructive pulmonary disease
f) heart failure

1. of the following fatty acids, please indicate those that you do not think belong to the group of essential fatty acids (EFAs)

a) polyunsaturated fatty acids
b) monounsaturated fatty acids
c) saturated fatty acids
d) I don't know

2. monounsaturated fatty acids in your opinion include the following

a) oleic acid
b) palmitic acid
c) lauric acid
d) I don't know

3. omega-3 and omega-6 acids, in your opinion, belong to the group of

a) saturated fatty acids
b) polyunsaturated fatty acids
c) monounsaturated fatty acids
d) I don't know

4. which of the following products do you think are sources of omega-6 fatty acids

a) lard
b) margarine
c) sunflower oil
d) peanut butter

5. which of the following products do you think is the richest source of omega-3 fatty acids

a) shrimp
b) mackerel
c) olive oil
d) cod
e) I don't know

6. sources of n-3 fatty acids (DHA and EPA) according to you are

a) fish
b) butter
c) lard
d) canola oil
e) I don't know

7 Which of the following functions do you think are attributed to essential fatty acids (EFAs)

a) anti-arrhythmic
b) anticoagulation
c) anti-atherosclerotic
d) anti-inflammatory
e) none of the above
f) all of the above
g) I don't know

8. what ratio of omega-6 to omega-3 fatty acids is optimal in your opinion

a) it doesn't matter
b) 1:1
c) 15:6
d) 5:1
e) I don't know

9. saturated fatty acids

a) increase the concentration of the LDL fraction to cholesterol
b) reduce the concentration of total cholesterol and LDL fraction
c) show no effect on blood cholesterol levels
d) I don't know

10. In your opinion, does a healthy lifestyle, proper diet and the type of fats consumed in the diet have a major impact on cardiovascular disease

a) yes
b) not
c) I don't know

11. in your opinion, can a diet rich in saturated fatty acids promote the development of cardiovascular diseases

a) yes
b) not
c) I don't know

12. the cause of increased mortality from cardiovascular diseases is

a) fat-rich diet
b) energy-rich diet
c) smoking
d) coexisting overweight/obesity
e) all of the above
f) I don't know

13. in your opinion, can a large consumption of animal products, rich in saturated fatty acids promote the development of atherosclerosis, and consequently ischemic heart disease

a) yes
b) not
c) I don't know

14. what products in your opinion are recommended for primary and secondary prevention of cardiovascular diseases

a) red meat
b) legumes
c) butter
d) fish
e) I don't know

15. diet in primary and secondary prevention of cardiovascular diseases should be based on

a) Increase intake of saturated fatty acids
b) Reduce the intake of saturated fatty acids and increase the supply of polyunsaturated fatty acids
c) Increasing the proportion of animal fats in the diet
d) I don't know

16. which of the following diets, is used in the prevention of cardiovascular disease

a) cabbage diet
b) Kaczynski's diet
c) A diet with a restriction of easily digestible carbohydrates
d) Mediterranean diet

17. mark the sentence false about the Mediterranean diet

a) Effective in preventing cardiovascular incidents
b) reduces serum cholesterol levels
c) Recommended for patients after myocardial infarction
d) A diet rich in protein and animal fats
e) I don't know

18.In the prevention of cardiovascular diseases, it is necessary to

a) Increase consumption of simple sugars, full-fat dairy products
b) limit consumption of fish and seafood
c) increase consumption of fish and nuts
d) I don't know

19. do you pay attention to the fat content of the product you choose

a) yes
b) not

20. in your opinion, does butter/margarine increase cholesterol levels

a) yes
b) not
c) I don't know

21. which of the following products contains the most cholesterol

a) egg white
b) yolk
c) skimmed cottage cheese
d) 18% cream

22. which type of fat dominates your diet

a) vegetable fats
b) animal fats

23. when buying cottage cheese, you most often reach for

a) skim quark
b) semi-skimmed curd
c) quark

24. which of the following fats do you use most often to fry a dish

a) margarine
b) butter
c) canola oil
d) olive oil

25. how often do you eat fried foods

a) I don't eat fried foods
b) once a week
c) several times a week
d) daily

26. how often do you eat fish

a) I do not consume
b) once a month
c) twice a week
d) once a week

27. the main risk factors for cardiovascular diseases in your opinion are as follows

a) dyslipidemia
b) hypertension
c) diabetes
d) obesity
e) none of the above
f) all of the above

28. in your opinion, is hypertension a disease for which fish oils (EPA and DHA) show effective improvement in hypotensive therapy

a) yes
b) not
c) I don't know

29 A diet rich in fish oils and fish, responsible for reducing cardiovascular incidents, is to

a) Increase the concentration of HDL fraction cholesterol and decrease the concentration of LDL fraction cholesterol
b) Increase in the concentration of LDL fraction cholesterol and decrease in the concentration of HDL cholesterol
c) Increased levels of LDL and HDL fraction cholesterol
d) Reduction of HDL and LDL fraction cholesterol levels

30. in your opinion, can regular consumption of fish and appropriate proportions of fatty acids in the diet contribute to reducing the risk of cardiovascular diseases

a) yes
b) not
c) I don't know
